# Prevalence of Uncorrected Refractive Error and Other Eye Problems Among Urban and Rural School Children

**DOI:** 10.4103/0974-9233.53864

**Published:** 2009

**Authors:** Amruta S. Padhye, Rajiv Khandekar, Sheetal Dharmadhikari, Kuldeep Dole, Parikshit Gogate, Madan Deshpande

**Affiliations:** Department of Community Ophthalmology, HV Desai Eye Hospital, Pune, India; British Columbia Center for Epidemiologic & International Ophthalmology, University of British Columbia, Canada

**Keywords:** Blindness, Refractive Error, School Health

## Abstract

**Background:**

Uncorrected refractive error is an avoidable cause of visual impairment.

**Aim:**

To compare the magnitude and determinants of uncorrected refractive error, such as age, sex, family history of refractive error and use of spectacles among school children 6-15 years old in urban and rural Maharashtra, India.

**Study Design:**

This was a review of school-based vision screening conducted in 2004-2005.

**Materials and Methods:**

Optometrists assessed visual acuity, amblyopia and strabismus in rural children. Teachers assessed visual acuity and then optometrists confirmed their findings in urban schools. Ophthalmologists screened for ocular pathology. Data of uncorrected refractive error, amblyopia, strabismus and blinding eye diseases was analyzed to compare the prevalence and risk factors among children of rural and urban areas.

**Results:**

We examined 5,021 children of 8 urban clusters and 7,401 children of 28 rural clusters. The cluster-weighted prevalence of uncorrected refractive error in urban and rural children was 5.46% (95% CI, 5.44-5.48) and 2.63% (95% CI, 2.62-2.64), respectively. The prevalence of myopia, hypermetropia and astigmatism in urban children was 3.16%, 1.06% and 0.16%, respectively. In rural children, the prevalence of myopia, hypermetropia and astigmatism was 1.45%, 0.39% and 0.21%, respectively. The prevalence of amblyopia was 0.8% in urban and 0.2% in rural children. Thirteen to 15 years old children attending urban schools were most likely to have uncorrected myopia.

**Conclusion:**

The prevalence of uncorrected refractive error, especially myopia, was higher in urban children. Causes of higher prevalence and barriers to refractive error correction services should be identified and addressed. Eye screening of school children is recommended. However, the approach used may be different for urban and rural school children.

## INTRODUCTION

Refractive error could be considered as an avoidable condition among various conditions leading to visual disabilities in children. Provision of spectacles to the needy is a cost-effective health intervention. Hence the VISION 2020 initiative to eliminate avoidable blindness has given high priority to correction of refractive error and has placed it within the category of “childhood blindness.”^1^ Most of the children with uncorrected refractive error are asymptomatic and hence screening helps in early detection and timely interventions. In countries with high attendance of children in schools, integration of vision screening within screening for other health issues is recommended.^2^ However, differences in the availability of access to eye care services and even the magnitudes of refractive error between rural and urban students are not considered.

Such a vision screening project was introduced in Maharashtra, India.^3^ The staff of community ophthalmology unit annually screens school children both in urban and rural areas. The children of remote rural areas are examined in mobile eye units, while the school teachers trained in vision screening examine students of urban schools. All children identified with refractive error are offered spectacles at low cost.

We conducted a study in two districts of Maharashtra as part of this screening campaign to compare the magnitudes and risk factors of uncorrected refractive error in 6 -15 years old school children of urban Maharashtra and those of rural Maharashtra. Based on the study results, we recommended policies for eye care of children in these age groups.

## MATERIALS AND METHODS

This study was conducted between August 2004 and July 2005. The ethical committee of the H.V. Desai Eye Hospital, Pune, India, gave written consent to conduct this study. Written consents of school principals of all selected schools and the Ministry of Education were obtained. Verbal consents of parents were obtained for screening their wards. The research protocol adhered to the provision of the Declaration of Helsinki for research involving human beings.

The urban clusters were selected according to their location in the electoral wards. We randomly selected 4 of the 48 electoral wards. These wards were Hadapsar, Indira Nagar, Bibewadi and Sahakarnagar of Pune Municipal Corporation. The rural clusters comprised of randomly selected 28 out of the 128 villages of Akalkot *taluka* (with 0.21 million population) of Solapur district.

To achieve 95% confidence limit and 90% of power in our study, having urban-to-rural ratio of 1:1.5, we used STATCAL of Epi Info 6(Centers for Disease Control and Prevention, Atlanta, GA, USA) to calculate the sample size for our study. By taking the reference of 5% prevalence of uncorrected refractive errors in urban students and 2.5% prevalence in rural students, it was determined that at least 1,061 randomly selected children in urban and 1,591 children in rural areas needed to be recruited for the study. To compensate for the effect of clustering at school levels, we inflated the sample by a factor of 2. Thus final sample size needed to compare the prevalence of uncorrected refractive error in urban and rural areas would be at least 2,122 children in the urban and 3,182 children in the rural group.

The study field staff included ten school teachers trained in vision screening, two optometrists, one ophthalmologist and one camp coordinator. Prior to the initiation of the study all field investigators were familiarized with the standard operating procedures involved. A pilot was conducted to validate the data collection forms to minimize inter-observer variations.

The field investigators obtained a detailed history about present and past ocular disorders, history of medical or surgical treatment and a family history of refractive error in the siblings. The distant vision of a child was tested utilizing Snellen's Illiterate ‘E’ chart. The visual acuity was tested at with the chart at 6 meters. If uncorrected vision was <6/12 in either eye, the child was declared to have defective vision.^2^ The Hirschberg's test was used to determine the presence or absence of strabismus. A cover-uncover test was then performed to confirm the diagnosis if strabismus was present. If eyes moved after removal of the cover, the child was considered to have a “phoria”; and if the degree of deviation did not change on cover and uncover, the child was considered to have a “tropia” [> 5 degree / 10Δ diopter (D)]. The eye movements were tested in 6 cardinal directions to rule out paralytic or restrictive strabismus. Anterior segment was examined with flashlight to detect cataract; congenital anomalies like anophthalmos, microphthalmos, large corneas; and evidence of previous eye surgery. In the presence of ocular pathology or symptoms of eyestrain, we performed fogging to rule out accommodative spasm. For fogging, we placed + 10 D lens in a trial frame and then gradually reduced the strength of the lens while the child continued to look at the eye chart. We performed streak retinoscopy using a + 1.5 D lens in the right eye frame and asking the child to fixate at a 6-meter distant target in order to relax accommodation. The children with visual acuity of 6/6 and with retinoscopic readings that confirmed the absence of a refractive error using the method described above were excluded from further refraction procedures. For other children after orthoptic and anterior segment evaluation, 1-2 drops of 1% Cyclopentolate eye drops were instilled twice over 20 minutes, and the cycloplegic refraction was performed using streak retinoscopy. The visual acuity, type of refractive error and correction was noted in children that were wearing spectacles.Myopia was considered when the measured objective refraction was more than or equal to –0.75 spherical equivalent diopters in one or both eyes. Hyperopia was considered when the measured objective refraction was greater than +2.00 spherical equivalent diopters in one or both eyes provided no eye was myopic. Astigmatism was considered to be visually significant if ≥1.00 D.

The data was entered in Microsoft XL^®^ spreadsheet after ensuring completeness of the filled forms. If any missing data was noticed, the concerned authority was contacted at the earliest and the details were rechecked. Analysis was done using the Statistical Package for Social Science (SPSS 10.0.5) (SPSS Inc. Chicago, USA). For assessing the magnitude of astigmatism, the readings of ≥ 2 D were taken into account; and for the readings < 2 D, their spherical equivalent was calculated by using the following formula:

Spherical equivalent = Spherical value + [cylindrical value / 2] (in diopters).^4^

We estimated the prevalence of uncorrected refractive error using parametric methods and univariate type of analysis. To validate the data, we calculated frequencies, percentage and their 95% confidence intervals. To compare the rates of urban and rural areas, we used odds ratio and chi-square values. As clustering of students was done in relation to schools, we performed weighted frequencies of different variables by school code. To study the effect of interaction of determinants on uncorrected myopia, we conducted multi-logistic regression analysis. Variables like age group, gender and area of residence were introduced in the model simultaneously.

All children with uncorrected refractive error were given spectacles at low cost. Children with eye disease were further examined and managed at the base hospital free of cost. The study results were shared with the scientific fraternity and policies for improving eye care of children were proposed.

## RESULTS

We evaluated 5,227 children that were randomly selected from 8 municipal schools,. We also evaluated 7,814 children who were randomly selected from 28 rural schools. We examined 12,422 children of ages in the range of 5 to 15 years. The coverage of screening was 5,021/5,227 (96.3%) in urban and 7,401/7,814 (94.8%) in the rural group. The characteristics of children examined in rural and urban groups were compared (Table 1). The urban and rural groups significantly differed by proportions of students in ‘age groups.’ We therefore determined that cluster-adjusted rates in urban and rural groups were needed before comparing the refractive error profile of urban and rural groups.

**Table 1:** Characteristics of 6 to 15 Years Old Children Examined in Urban and Rural Schools

Variant		Urban		Rural		Validation
			
		Number	%	Number	%	
Sex	Male	2,939	58.5	4,314	58.3	OR = 1.01
	Female	2,082	41.5	3,087	41.7	95% CI 0.94 to 1.09
Age group	6 to 8 yrs	1,600	31.9	1,597	21.6	χ^2^ = 540.1
	9 to 12 yrs	2,536	50.5	3,102	41.9	Df = 2
	13 to 15 yrs	885	17.6	2,702	36.5	*P* <0.0001
F/H of refractive error in siblings	Yes	2	0.04	0	0.0
	No	5,019	99.96	7,401	100.0	
Children using spectacles	Yes	10	0.2	9	0.1	OR = 1.62
	No	5,011	99.8	7,392	99.9	95% CI 0.62 to 4.38
Total		5,021	100	7,401	100	

OR: odds ratio, CI: Confidence intervals, Df: Degree of freedom

Uncorrected refractive errors were the reason for defective vision in 141 (77%) and 121 (90%) students of urban and rural areas, respectively. The cluster-adjusted prevalence of uncorrected refractive error among urban and rural children was 5.46% (95% CI, 5.44-5.48) and 2.63% (95% CI, 2.62-2.64), respectively. The cluster-adjusted prevalence of myopia, hypermetropia and astigmatism in urban children was 3.16%, 1.06% and 0.16%, respectively. In rural children, the cluster-adjusted prevalence of myopia, hypermetropia and astigmatism was 1.45%, 0.39% and 0.21%, respectively. The frequencies, adjusted rates, their 95% confidence intervals were compared for children of rural and urban areas and for the subgroups like gender and age group (Table 2).

**Table 2:** Prevalence of Uncorrected Refractive Error in Children of Urban and Rural Schools

	Urban				Rural				Validation
		
	Examined	Uncorrected refractive error	Rate*	95% CI	Examined	Uncorrected refractive error	Rate*	95% CI	
Male	2,939	155	5.27	5.25 5.30	4,314	92	2.12	2.6	OR = 1.09
Female	2,082	119	5.72	5.69 5.75	3,087	104	3.35	2.7 - 4.0	(0.85 to 1.40)
6 to 8 yrs	1,600	50	3.13	3.10 3.15	1,597	15	0.93	0.47 -1.41	χ^2^ = 57.7
9 to 12 yrs	2,536	183	7.22	7.18 7.25	3,102	90	2.9	2.31 -3.49	Df = 2
13 to 15 yrs	885	41	4.63	4.59 4.67	2,702	90	3.3	2.65 -4.01	*P* < 0.0001
Myopia	5,021	160	3.2	2.70 3.67	7,401	108	1.5	1.19 - 1.73	
Hyperopia		53	1.1	0.77 1.34		29	0.4	0.25 - 0.53	
Astigmatism		8	0.2	0.05 0.27		16	0.2	0.11 -0.32	
Total	5,021	274	5.46	4.83 6.09	7,401	196	2.65	2.28 - 3.01	OR = 2.06 (1.72 - 2.47)

* The prevalence proportions are cluster adjusted. OR: odds ratio, CI: Confidence intervals, DF: Degree of freedom

We also calculated crude rates of uncorrected myopia and hypermetropia in urban and rural groups in different age groups (Table 3). The prevalence of myopia varied significantly among urban and rural students of different age groups.

**Table 3:** Prevalence of Uncorrected Refractive Error by Type and Age Group in Children of Urban and Rural Schools

	Urban				Rural				Validation
		
	Examined	Uncorrected Myopia	Rate*	95% CI	Examined	Uncorrected refractive error	Rate*	95% CI	
Myopia									
6 to 8 yrs	1,600	23	1.4	0.9 - 2.0	1,597	6	0.4	0.1 - 0.7	χ^2^ =45.8
9 to 12 yrs	2,536	107	4.2	3.4 - 5.0	3,102	39	1.3	0.9 - 1.6	Df = 2
13 to 15 yrs	885	30	3.4	2.2 - 4.6	2,702	63	2.3	1.8 - 2.9	*P* < 0.0001
Hypermetropia									
6 to 8 yrs	1,600	15	0.9	0.5 - 1.4	1,597	6	0.4	0.1 - 0.7	χ^2^ =0.67
9 to 12 yrs	2,536	31	1.2	0.8 - 1.7	3,102	18	0.6	0.3 - 0.8	Df = 2
13 to 15 yrs	885	7	0.8	0.2 - 1.4	2,702	5	0.2	0.02 - 0.3	*P*= 0.71

* Prevalence proportion

We conducted multivariate regression analysis to determine the predictors of uncorrected myopia (Table 4). Being a resident of urban area (OR = 2.54) and belonging to the 6 to 9 years age group (OR = 0.27) were the predictors of uncorrected myopia.

**Table 4:** Uncorrected Myopia in 6 to 15 Years Old Children and Its Predictors

Variant		Adjusted Odds Ratio*	95% Confidence Interval	*P* value
Intercept		-3.93		
Gender	Male	0.98	0.76 - 1.25	0.86
	Female	1.0		
Area of residence	Urban	2.54	1.97 - 3.27	6.9 × 10^−13^
	Rural	1		
Age group	6 to 9	0.27	0.17 -0.41	1.2 × 10^−9^
	10 to 12	0.81	0.62 - 1.07	0.13
	13 to 15	1		

Amblyopia was found in 41 (0.8%) children of urban schools and 17 (0.23%) children of rural schools. The amblyogenic factors among children with amblyopia were also noted. In the urban group, 33 (0.7%) children had isoametropic, anisometropic and meridional refractive error, 6 (0.1%) children had strabismus and 2 (0.04%) had strabismus associated with high refractive error. In contrast, amblyopia was noted in 16 (0.22%) children with refractive errors and 1 (0.01%) child with strabismus among rural children.

The causes of vision impairment in children of urban and rural groups were evaluated. We found 264 children with uncorrected refractive error, 41 with amblyopia, 4 with cataract, 6 with corneal opacities, 4 with disease of retina, 1 with strabismus and 4 with other [phthisical, microcornea, nystagmus] disorders among the urban group. While in rural group, we detected 187 children with uncorrected refractive error, 17 with amblyopia, 2 with cataract, 2 with corneal opacities and 2 with diseases of retina.

In the rural group, 9 (4.6%) out of 196 children with refractive error were using spectacles. Ten (3.65%) out of 274 children with refractive error in the urban schools were using spectacles at the time of examination.

## DISCUSSION

The prevalence of uncorrected refractive error, especially myopia, was significantly higher (OR = 2.06) in 6 to 15 years old school children of urban area when compared to children of rural schools. The coverage of refractive services was uniformly poor in both urban and rural areas. We also found presence of blinding diseases such as cataract in 6 children, corneal opacities in 8 children, both in urban and rural areas. Although strabismus was detected in few children, amblyopia was detected in 58 children during school screening. Children with amblyopia need timely treatment, otherwise amblyopia could become dense and irreversible.^5^ Our study suggested that the likelihood of detecting uncorrected myopia was higher in older students and that the chances of detecting uncorrected myopia was higher in students living in rural areas..

Among school children in the 2 study areas, we focused on uncorrected refractive error rather than the magnitude and risk factors of refractive error. We found that even after accounting for the differences in rates by age groups and gender, the prevalence rates of myopia were significantly different between school children that lived in rural and urban areas. One could speculate that this difference could be influenced by a combination of the following factors: (1) the screening methods used in urban and rural areas, (2) the uptake of refractive services in these 2 areas, (3) the difference in prevalence rates of refractive error and other eye conditions between children of rural and urban areas. Proper methods with quality checks applied in our study ensured that differential methods had minimum influence on identification of children with uncorrected refractive error. In our study, we found that 4.6% of rural and 3.65% of urban children with refractive error used spectacles. Thus uptake of refractive services in the past does not seem to make a significant difference. Therefore, rates and the risk factors of refractive error among urban and rural children truly seem to be different.

The higher prevalence of uncorrected refractive error in urban areas of our study matched with the observations made in Delhi and Andhra Pradesh studies.^6^,^7^ It should be noted that all these 3 studies used similar protocols. The prevalence of uncorrected refractive error in urban areas in our study was similar to the 5.65% reported in another study conducted in the past in Pune.^8^ Sethi *et al.* and Matta *et al.* also observed that urban children had higher risk of developing refractive errors.^9^,^10^

Myopia was the main type of uncorrected refractive error in our study. The rate of myopia was significantly higher in urban children compared to rural children. A comparative study by Dandona *et al.* found prevalence of myopia to be 5% and 2.5% in urban and rural regions, respectively, of Andhra Pradesh.^11^ However, lower prevalence of myopia in our study could be due to different definitions of myopia used in the above-mentioned studies (< –0.50 D). Khandekar *et al.* in Oman and Morgan *et al.* in rural Mongolia noted myopia prevalence of 4.1% and 5.8%, respectively among school children,.^12^,^13^ In spite of having different races in the 3 study areas, the rates were similar.

The prevalence and severity of myopia were significantly higher in children of urban schools compared to those of rural schools in Taiwan.^14^ Lithander found that the prevalence of high myopia (≥ 7.0 D) among 12-year-old girls was 2.82% compared to 0.13% prevalence in the general population. This could be due to genetic predisposition for high myopia among either females or children of semi-urban towns of Oman.^15^ In our study, we did not observe myopia of such large magnitude among urban girls. Dandona *et al.* in Andra Pradesh Eye Diseases Study (AEPDS) study also noted that urban location was a predictor of myopia, and children of urban area had 2.5 times higher risk compared to rural children.^16^ Saw *et al.* in a study in Xiamen province of China, noted that 2^nd^ grade students of urban area had myopia at the rate of 19.3% (95% CI, 12.3-29.0). Among children of rural area, it was 6.6% (95% CI, 2.4-14.3). Although myopia in urban students was linked to more near work compared to the students of rural area, element of chance in this observation could not be ruled out.^17^ Prevalence of myopia was 2.9% in Sherpa (nomadic) children, while Tibetan children (settled in Nepal) had myopia at the rate of 21.7%.^18^ Thus different studies suggest that myopia seems to be significantly associated to the life style in urban areas. While comparing outcomes of different studies, one should remember that the prevalence identified in studies on school-going children would be higher than that identified in studies on school-aged populations.

Czepita *et al.* noted that gender influences the occurrence of myopia and hyperopia in school-going children of age ranging from 6 to 18 years.^19^ Therefore, while studying the magnitude of uncorrected refractive error in relation to rural and urban population, we conducted regression analysis. It is interesting to note that when we conducted univariate analysis, gender was not significantly associated to uncorrected refractive error in urban and rural children; however, boys had higher risk of uncorrected refractive error after accounting for other confounders. This issue could be further studied by conducting a longitudinal study.

Our study had few limitations. There might be an underestimation of all refractive errors as cycloplegia was performed only for the children that had retinoscopy readings suggestive of myopia > –2.00 D, hyperopia > +0.50 D, astigmatism > 1.00 D. We attempted to address accommodative spasm by utilizing the fogging method. However, accommodative spasm could not be entirely ruled out.

General practitioners,^20^ school nurses,^12^ teachers^21^ and optometrists^22^ have been involved in eye and vision screening in different studies. In our study, we involved teachers of urban schools and trained them in vision-testing procedures. After they screened the children, optometrists rechecked the findings. On the other hand, the same optometrist conducted vision screening of students of rural schools during their visits to the vision center. Both approaches were feasible, and even the coverage and quality of screening was high. Thus irrespective of the manpower used, if proper checks are in place, vision screening can be implemented and refractive error among school children can be addressed. However, sustainability of such a different approach should be studied before recommending it to others to adopt it as a system.

Vision screening and refractive services for school students have been recommended by WHO.^2^ The magnitude and causes of uncorrected refractive error seem to differ in urban and rural areas of India. Hence refractive services should be adapted to the situation in the various areas of developing countries.
